# Profiling molecular and behavioral circadian rhythms in the non-symbiotic sea anemone *Nematostella vectensis*

**DOI:** 10.1038/srep11418

**Published:** 2015-06-17

**Authors:** Matan Oren, Ann M. Tarrant, Shahar Alon, Noa Simon-Blecher, Idan Elbaz, Lior Appelbaum, Oren Levy

**Affiliations:** 1 The Mina & Everard Goodman Faculty of Life Sciences, Bar-Ilan University, Ramat-Gan 52900, Israel; 2 Biology Department, Woods Hole Oceanographic Institution, Woods Hole MA 02543, USA; 3 George S. Wise Faculty of Life Sciences, Department of Neurobiology, Tel-Aviv University, Tel-Aviv 69978, Israel; 4 Sagol School of Neuroscience, Tel-Aviv University, Tel-Aviv 69978, Israel; 5 The Leslie and Susan Gonda Multidisciplinary Brain Research Center, Bar-Ilan University, Ramat-Gan, Israel

## Abstract

Endogenous circadian clocks are poorly understood within early-diverging animal lineages. We have characterized circadian behavioral patterns and identified potential components of the circadian clock in the starlet sea anemone, *Nematostella vectensis*: a model cnidarian which lacks algal symbionts. Using automatic video tracking we showed that *Nematostella* exhibits rhythmic circadian locomotor activity, which is persistent in constant dark, shifted or disrupted by external dark/light cues and maintained the same rate at two different temperatures. This activity was inhibited by a casein kinase 1δ/ε inhibitor, suggesting a role for CK1 homologue(s) in *Nematostella* clock. Using high-throughput sequencing we profiled *Nematostella* transcriptomes over 48 hours under a light-dark cycle. We identified 180 *Nematostella* diurnally-oscillated transcripts and compared them with previously established databases of adult and larvae of the symbiotic coral *Acropora millepora,* revealing both shared homologues and unique rhythmic genes. Taken together, this study further establishes *Nematostella* as a non-symbiotic model organism to study circadian rhythms and increases our understanding about the fundamental elements of circadian regulation and their evolution within the Metazoa

The phrase “timing is everything” is often accurate. Since the beginning of life on this planet, organisms have evolved under periodic cycles of light and temperature, caused by the Earth’s rotation and revolution. In response to these cyclic changes, endogenous clocks have evolved in many organisms, allowing them to anticipate daily and seasonal environmental rhythms and to adjust their biochemical, physiological, and behavioral processes accordingly^1^,^2^. The most widely studied endogenous biological clock is the circadian clock, an endogenous self-sustained system that drives daily physiological and behavioral rhythms. Broadly, circadian clocks are built from three components: 1) environmental sensors in the clock input pathway through which entraining signals from the environment (e.g., light and temperature) are perceived, 2) transcriptional-translational feedback loops in the core oscillator, which maintain the clock pacing and transmit rhythmic signals to downstream components^3^ and 3) clock-controlled genes (CCGs), which respond to core oscillator pacing signals and coordinate circadian responses within cells^4^. In addition,post-translational mechanisms, such as phosphorylation of PERIOD proteins in bilaterian animals by casein kinase 1 family members, are also involved in the clock regulation^5^. Circadian clocks have been characterized in cyanobacteria, fungi, plants, and animals; however, there is little conservation in clock pathway architecture among these different taxonomic groups^6^, indicating that circadian rhythmicity is a key adaptive element that evolved independently in metazoans and in several non-metazoan groups^7^. Within the bilaterian animals, a great deal has been learned about circadian signaling through studies conducted in well-characterized model organisms. Through such studies, investigators have identified both components that are shared among bilaterian animals and those that are restricted to specific lineages. However, findings in these earlier studies also indicate that every model system has its own set of adaptations, specializations, and caveats^6^,^8^. Thus, to further expand our understanding of the evolutionary history of circadian behavior and rhythmic gene expression, study of these processes in species that diverged at informative points in evolution are required.

Cnidarians are ecologically important marine and aquatic organisms that arose about 740 million years ago^9^ and possess a worldwide distribution. They are the simplest extant animals to possess a true tissue-grade of organization (Eumetazoa) and are particularly informative in making inferences about the gene content of the common metazoan ancestor^10^. An understanding of rhythmic regulation of behavior in cnidarians would provide insight both into the evolution of animal circadian clocks and into the physiology of this key animal group.

The starlet sea anemone, *Nematostella vectensis*, has emerged as a powerful cnidarian model with a sequenced genome and a growing suite of available molecular resources and tools^11^,^12^. *Nematostella* is widely distributed in brackish environments and unsurpassed for the ease with which its entire life cycle is maintained in the laboratory^13^,^14^. As proof of its utility, *Nematostella* has already provided a first glance into the evolution of the metazoan circadian clock^15^,^16^. Several recent studies have indicated that *Nematostella* and reef-building corals share homologues of some core clock genes with bilaterians^15^,^17^,^18^,^19^. In addition, microarray studies of the coral *Acropora millepora* have identified groups of genes including antioxidants, metabolic enzymes, and chaperones that exhibit daily oscillations in expression and may be regulated by circadian mechanisms^20^. However, many questions remain regarding the mechanism of circadian regulation as well as physiological and behavioral significance of the circadian clock in cnidarians. While *Acropora* and *Nematostella* are both members of the class Anthozoa, they exhibit substantial physiological differences. In particular, *Acropora* and other reef-building corals typically host algal symbionts, which are likely to possess their own circadian clocks and which introduce strong diurnal metabolic signals associated with photosynthesis^21^. Because *Nematostella* lacks algal symbionts, it provides a simpler cnidarian model of circadian regulation.

Here we have characterized the *Nematostella* circadian locomotor activity using a video tracking system under light dark cycles (LD) and under free- running conditions of constant darkness (DD) and constant light (LL). In addition, we have demonstrated that selective inhibition of casein kinase signaling disrupts the circadian locomotor activity under DD free-running conditions. Finally, to characterize the molecular rhythmic actors of *Nematostella*, RNA-seq and whole transcriptome analysis were conducted during day and night.

## Results

### *Nematostella* locomotor activity is rhythmic and is controlled by endogenous circadian clock

**Figure 1:**
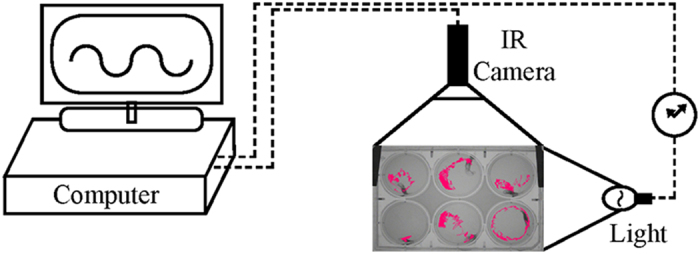
*Nematostella* locomotor activity tracking. Adult *Nematostella* were separated in wells of six-well plates and were constantly monitored by an infrared camera inside the Noldus DanioVision XT tracking device. White light was automatically turned on and off as required during the 72–108 hours of the experiments. The red dots within the wells’ arenas illustrate the movement paths of the six anemones. Significant differences in the total distance moved were recorded between individuals; therefore, we used the ratios from the maximal value recorded for each animal in each of the experiments.

**Figure 2:**
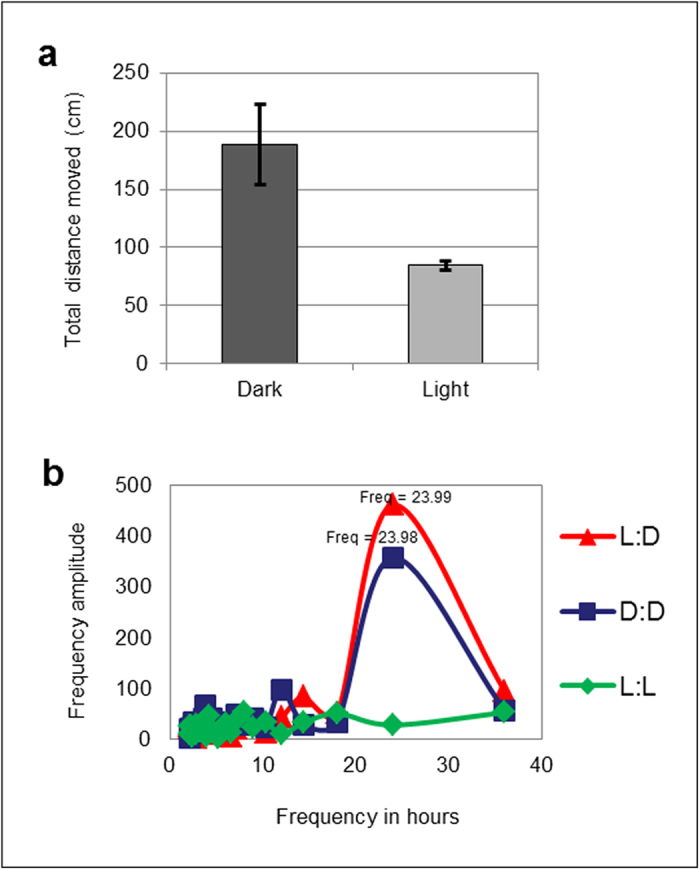
*Nematostella* locomotor activity shows circadian oscillations with nocturnal maxima. (**A**). The average total distance moved in dark hours (ZT12–ZT24) was 188.6 cm (n = 35; SE = 34.3) compared to 84.5 cm (SE = 4.1) during light hours (ZT0–ZT12). (**B**). In the LD and DD experiments, the major oscillation frequency peak identified through Fourier analysis is almost exactly 24 h. In the LL experiment no significant oscillation frequency was identified.

**Figure 3:**
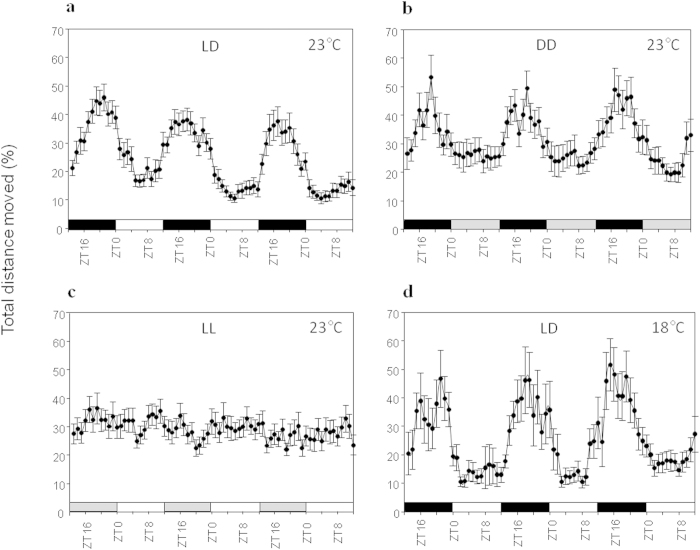
*Nematostella* locomotor activity has an endogenous rhythm. (**A**). *Nematostella* locomotor activity under a 12 : 12 h light : dark cycle (LD) at 23 °C. (**B**). *Nematostella* locomotor activity in constant dark (DD) at 23 °C. (**C**). *Nematostella* movement in constant light (LL) at 23 °C. (**D**). *Nematostella* locomotor activity in LD at 18 °C. White bars indicate light hours, black bars indicate dark hours, and gray bars indicate illumination conditions different from an LD cycle (dark instead of light; light instead of dark).

The behavioral rhythms of *Nematostella* were studied by monitoring the locomotor activity of individuals using a tracking system, which was equipped with an infrared (IR) camera and time-controlled white LED illumination that can be set to different intensities (Fig. 1). In parallel with the locomotor activity tracking, some of the experiments were recorded with a video camera in order to enable visualization of the different movement patterns. Three major movement types were recorded (Supplementary Video S1): head movement from side to side, body banding and constant peristaltic movement along the body axis. Behavioral rhythms were initially characterized over 3 days under 12 h light: 12 h dark (LD) conditions at 23 °C. Automated infrared tracking showed that *Nematostella* exhibits greater locomotor activity during the subjective night. Under LD conditions, during the night (ZT12–ZT24), the tested animals (n = 35) moved a total of 188.6 cm on average (standard error (SE) = 34.3), compared to only 84.5 cm on average (SE = 4.1) during light hours (ZT0–ZT12; Fig 2A). The averaged *Nematostella* locomotor activity peaked between four and nine hours after dark onset (ZT16–ZT21). Within this time period, the animals moved on average 101.9 cm (SE = 20.8) compared to only 16.3 (SE = 3.9) in the equivalent time period during light hours (ZT4–ZT9). Fourier analysis of the locomotor activity average ratios during the three days in LD conditions resulted in a single significant periodogram peak at 23.99 h (n = 35), indicating circadian frequency (Fig. 2B in red). To normalize the differences in the absolute distance covered between *Nematostella* individuals that may originate from differences in size or metabolic rate, we have calculated the relative locomotor activity as a percentage of the maximum locomotor activity recorded for each animal (in LD the average relative locomotor activity ranged between a minimum of 11.2% during light hours and a maximum of 46% during dark hours, Fig. 3A). Similar to the LD results, a single significant frequency peak at 23.98 h was identified in constant dark free-running conditions (DD; n = 20; Fig. 2B in blue) with average relative locomotor activity ranging from 19.9% to 53.4% and following the same oscillation pattern as in LD (Fig 3B). These results support the existence of endogenous clock oscillator; however, in contrast with previous observation [16], our results didn’t show any significant circadian oscillation frequency during the constant light free-run (LL; n = 30; Fig. 2B in green). Under LL conditions, the average relative locomotor activity ranged from 22.2% to 32.1% with no significant dominant frequency (Fig. 3C).

As a first approach to study whether *Nematostella* oscillator exhibits temperature compensation, we inquired if the rhythms observed in LD conditions were also maintained at a lower temperature, we monitored the locomotor activity during three days under LD conditions at 18 °C (5 °C below the temperature of all other experiments). We observed a similar locomotor activity oscillation pattern as in the LD and DD experiments with locomotor activity ratios averages of 10.4% to 55.7% (Fig. 3D). The tested approach may have some limitations associated with potential masking effects of light. Nevertheless, the obtained results suggest potential temperature compensation of *Nematostella* circadian system.

### The *Nematostella* locomotor activity cycle can be shifted by dark pulse or disrupted by light pulse

**Figure 4:**
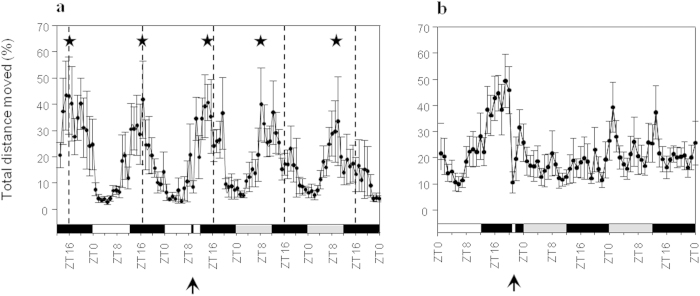
Activity phase shift and cycle disruption following dark or light pulses. (**A**). Locomotor activity oscillation phase is shifted following one hour of darkness (dark pulse) between ZT9 and ZT10 (of the second experiment day). (**B**). Locomotor activity oscillation is completely disrupted following 1 h of light pulse between ZT21 and ZT22 (of the first experiment day). White bars indicate light hours, black bars indicate dark hours, and gray bars indicate illumination conditions different from an LD cycle (dark instead of light; light instead of dark), stars indicate the locomotor activity peak, arrows indicate light/dark pulse.

We tested the effect of 1 h dark and 1 h light pulses on the locomotor activity oscillation. The pulses were performed during normal LD conditions, while the effect was tested under DD free-run in order to prevent entrainment by a light cue. When a 1 h dark pulse was applied between ZT9 and ZT10 (2 h before the entrained dark onset), the oscillation phase was advanced and changes were observed in the cycle length. The observed locomotor activity cycle length (based on peak locomotor activity) was advanced by 2 h on the next day (ZT14), by 8 h on the second day after the pulse (ZT8) and by 6 h on the third day after the pulse (ZT10) (n = 11; Fig. 4A). In contrast, a 1 h light pulse, between ZT21 and ZT22 (2 h before the entrained light onset) caused a complete disruption of the locomotor activity cycle during the following dark free-run for the rest of the experiment (Fig. 4B). Due to the nature of the experimental system, prolonged behavioral monitoring was not possible, so it is not possible to determine whether the observed disruption is transient or permanent.

### Rhythmic locomotor activity is inhibited by a pan-CK1δ/ε inhibitor, but not by a CK1δ-selective inhibitor in *Nematostella*

The casein kinase I (CK1) family consists of serine/threonine protein kinases, some of which are key regulators of circadian timing in bilaterian animals, fungi and green algae^22^. CK1-like genes have previously been identified in both *Acropora* and *Nematostella* and were suggested as components of circadian gene network in these organisms^23^. Reciprocal BLASTx searches of human and *Drosophila* CK1 sequences against predicted proteins in the *Nematostella* JGI genomic database revealed six CK1 family members in *Nematostella.* Three of the *Nematostella* CK1 sequences grouped into a clade with *Drosophila Doubletime* as well as human *CK1δ* and *CK1ε* (NvCK1_12115, NvCK1_12051, NvCK1_88486) Two others (NvCK1_159193 and NvCK1_161273) grouped with *Drosophila CK1* and human *CK1α* genes, and the final *Nematostella* gene (NvCK1_192152) grouped with human *CK1γ1* and *CK1γ3* (Supplementary Fig. S1).

To investigate a potential role for CK1 activity in circadian function in *Nematostella,* we characterized the effects of two specific pharmacological inhibitors of vertebrate CK1 activity on circadian behavioral rhythms in *Nematostella*. One of these inhibitors (PF-4800567) specifically targets CK1δ. The second (PF-670462) inhibits both CK1δ and CK1ε and has been shown to disrupt behavioral rhythms in distantly related organisms, such as the green alga *Ostreococcus tauri*^22^. The two inhibitors were tested at concentrations that have been shown to specifically inhibit circadian function in zebrafish^24^.

**Figure 5:**
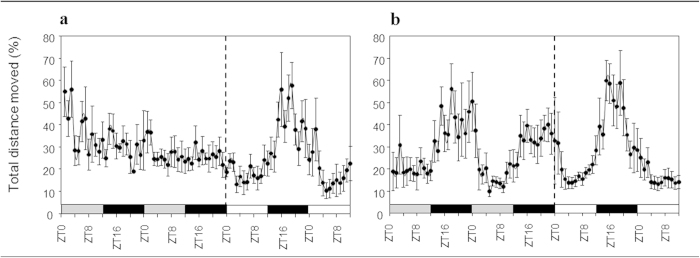
Inhibition of *Nematostella* locomotor activity oscillation by a CK1δ/ε inhibitor. **A**. *Nematostella* locomotor activity was inhibited during DD free-run in water containing 1 μM pan-CK1δ/ε inhibitor. Dashed line indicates initiation of recovery following the replacement of the water medium and shifting back to LD illumination regime. **B**. *Nematostella* locomotor activity in water containing 1 μM CK1δ inhibitor in the same conditions as in A. No changes in locomotor activity were observed.

Twelve hours prior to the initiation of the locomotor tracking, *Nematostella* individuals were incubated in 1 μM of the pan-CK1δ/ε inhibitor or CK1δ-selective inhibitor. Over the next two days (48 h), locomotor activity tracking was performed under DD free-running conditions followed by inhibitor-free recovery of 1.5 days (36 h) under LD conditions. CK1δ/ε inhibitor-treated *Nematostella* lost their locomotor activity oscillation (n = 12, Fig. 5A), while CK1δ inhibitor-treated *Nematostella* maintained their original oscillation. (n = 12, Fig. 5B). The locomotor activity oscillation of CK1δ/ε inhibitor-treated *Nematostella* was successfully recovered after replacing the water with inhibitor-free water and changing the light conditions back to LD (Fig. 5A). This suggests that one or more CK1 family members may be involved in the regulation of circadian behavior in *Nematostella.*

### Expression of many *Nematostella* genes exhibit diel rhythmicity

**Figure 6:**
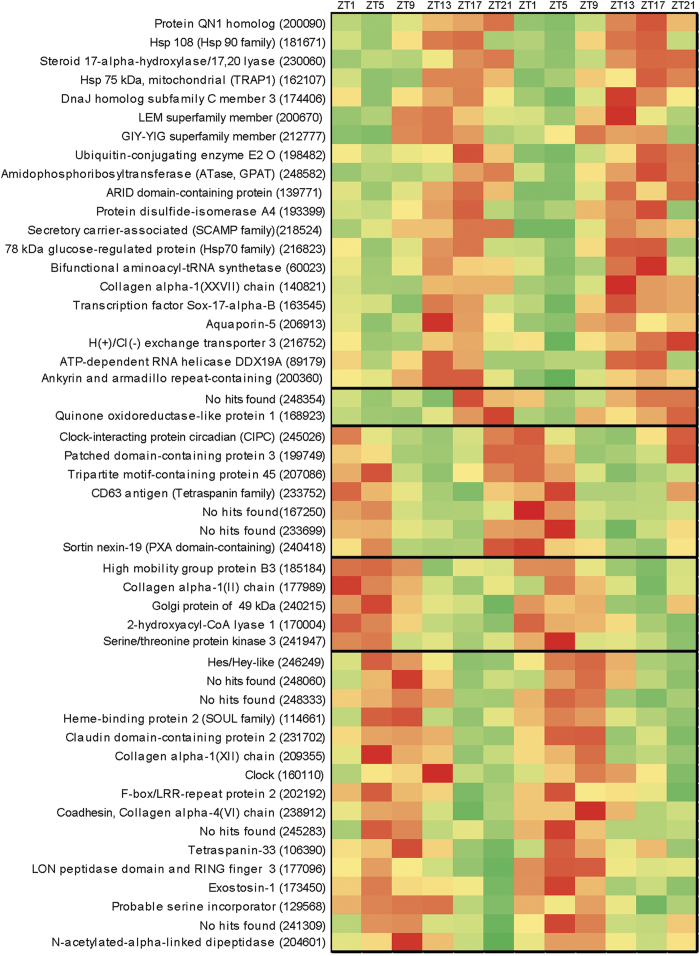
Heat map showing major expression of the 50 transcripts with strongest diel rhythmicity (highest g-factor) in *Nematostella*. Provisional annotation was based on the top hit to the Swissprot database. Some gene names were edited based on phylogenetic analysis of the *Nematostella* genes, and some family or domain names were added parenthetically. See supplementary Table S1 for more complete annotation. Color scale ranges from red to green (highest to lowest relative expression). The x-axis indicates time of sampling, where Zeitgeber time (ZT) is the number of hours since the light cue was turned on (lights were turned on at 7 am and off at 7 pm; 8 am is ZT1). Heavy black lines within the heat map indicate genes with similar expression patterns, as identified through K-means clustering.

To better understand the molecular forces that regulate the circadian locomotor activity rhythm in *Nematostella,* we conducted transcriptional profiling using the Illumina HiSeq platform with samples collected every four hours over two days under LD conditions identical to those in the behavioral assay (BioProject accession number: PRJNA246707). Using Fourier analysis, the possible diel rhythmicity (i.e., 24-h periodicity) of all the genes was quantified, and the 180 transcripts exhibiting a g-factor >0.5 were further analyzed. Through K-means clustering, these transcripts were divided into 5 groups, each with a characteristic peak expression time. The 50 transcripts exhibiting the strongest diel rhythm are shown in Fig. 6, and expression data for all 180 genes are listed in Supplementary Table S1; we subsequently refer to these as diel cycle genes (DCGs). Because these genes were identified based on their oscillations under LD conditions they were characterized as “diel control genes” (DCGs) rather than as “clock-controlled genes” (CCGs), which have been specifically demonstrated to maintain a cycle under constant conditions.

We annotated 143 of the DCGs through BLASTp-based searches of the SwissProt database. In addition, we identified putative homologues for 59% (22/37) of the unannotated genes through BLAST searches of the *Acropora millepora* genomic database. These may represent taxonomically restricted genes. GO terms were associated with 135 of the DCGs; however, none of these GO terms were statistically enriched in comparison with the *Nematostella* transcriptome.

In the present study, *NvClock*, *NvCry1a, NvCry1b* and *NvCry2* exhibited diel periodicity with similar timing of peak expression to that reported by Reitzel *et al.*^15^ (Table 1). Specifically, *NvClock* expression peaked late in the day (ZT9-13), *Cry1a* and *Cry1b* peaked during mid-day (ZT4-11 and ZT5-9, respectively), and *Cry2* peaked during early morning or late night (ZT0-4 in^15^ and ZT21 in the present study). Both *Clock* and cryptochromes play central roles in regulating circadian cycles in bilaterians. Some cryptochromes are light sensitive and act to directly coupling the circadian clock with exogenous light cues^25^.

### Comparative transcriptomic analysis reveals diel cycle genes shared between *Nematostella* and corals

*Nematostella* and the scleractinian coral *Acropora millepora* are both anthozoan cnidarians, but they differ profoundly in terms of habitat and symbiont composition. Individual *Nematostella* polyps lack algal symbionts and colonize salt marsh environments, while *A. millepora* forms calcified colonies on tropical reefs through an obligate symbiosis for dinoflagellates. Genes with circadian expression patterns in both taxa are likely to serve fundamental roles in circadian physiology of cnidarians.

**Figure 7:**
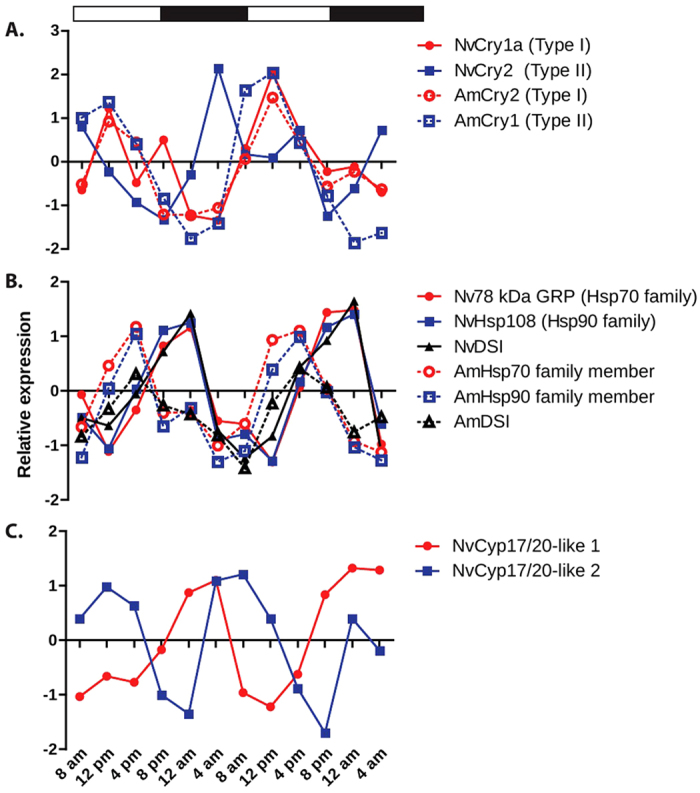
Temporal expression patterns of selected genes exhibiting a rhythmic periodicity in *Nematostella*. Where available, expression patterns of corresponding genes from *Acropora millepora* are shown using the same colors with dotted lines and open symbols. Expression values were standardized to facilitate visualization of genes with different expression levels on the same axes. (**A**) Type I cryptochromes (*NvCry1a* and *AmCry2*, red symbols) exhibit peak expression around noon in both species. Type II cryptochrome expression (*NvCry2*, *AmCry1*, blue symbols) peaks during subjective night in *Nematostella* (4 am) and during the day (noon) in *A. millepora.* Accession numbers in Table 2. (**B**) Heat shock proteins and disulfide isomerase oscillate strongly both in *A. millepora* and *Nematostella*, but the timing differs between the two taxa. For brevity, the heat shock proteins are labeled as *Hsp70* and *Hsp90* because they are members of these large families. See text for additional discussion. Accession numbers in Table 2. (**C**) Two cytochrome P450 genes that are most closely related to the CYP17/20 family exhibit diel oscillations that are out of phase with one another (JGI Accession numbers: *Cyp17/20-like 1*: 230060, *Cyp17/20-like 2*: 164939).

We first compared the set of 180 *Nematostella* DCGs with a set of CCGs that were identified from a previous microarray-based study of *Acropora millepora*^20^. Of note, the *A. millepora* genes exhibited daily oscillations both under LD conditions and under DD free-run. We mapped the differentially expressed microarray probes to 99 unique *A. millepora* transcripts, 9 of which are putative homologs of *Nematostella* DCGs (Table 2). Among the shared genes (*Nematostella* DCGs and *A. millepora* CCGs) were two cryptochromes. *Nematostella* and *A. millepora* each contain two Type I cryptochromes and one Type II cryptochrome. In each species, the Type II cryptochrome and one of the Type I cryptochromes exhibited diel oscillations (i.e., were identified as DCGs in *Nematostella* and as CCGs in *A. millepora,* Fig. 7a). The *Acropora* Type I and Type II cryptochromes have a similar oscillation pattern, which generally overlaps with a *Nematostella* Type I cryptochrome (*NvCry1a*), peaking at 12 pm (ZT18) but not with *Nematostella* Type II cryptochrome (*NvCry2*), which peaks at 4 pm (ZT10; Fig. 7a).

Additional genes that exhibited diel oscillations in both species were two heat shock proteins (members of the Hsp70 and Hsp90 families) and *protein disulfide isomerase*, all of which act as chaperones to maintain correct protein folding. In *A. millepora*, expression of these three genes peaked at 4 pm (ZT10), which was hypothesized to correspond to diel patterns of stress^20^. In contrast, in *Nematostella*, all three genes exhibited peak expression during subjective night (12 am, ZT17, Fig. 7b).

### Four additional diurnally oscillated genes in *Nematostella* and *A. millepora*

Four additional genes exhibited diel oscillations in both *Nematostella* and *A. millepora*: *Hes/Hey-like*, a heme-binding protein in the SOUL family, a high mobility group B protein (HMGB), and a transcript with no similarity to genes of known function. The unannotated gene did exhibit significant similarity (40–50% amino acid identity, e-values around 1 × 10^−40^) to predicted proteins of unknown function from diverse metazoans. *Hes/Hey-like* exhibited a strong diel expression pattern in both species, with peak expression at noon for *Nematostella* and 8 am for *A. millepora*. Shoguchi *et al.*^26^ showed that *Nematostella Hes/Hey-like* falls within a clade of basic-helix-loop-helix transcription factors that contain an orange domain (bHLH-O). The SOUL family member exhibited similar expression in both species (daytime maxima), but the HGMB and unannotated gene did not.

The *Nematostella* transcripts that exhibited diel oscillations in expression were also compared with an Illumina-based study of gene expression during the day and night in *Acropora millepora* larvae^19^. Of the 180 *Nematostella* genes with diel oscillations in expression, we identified putative homologues of 108 genes within the *A. millepora* data set. Six of these *A. millepora* transcripts exhibited ≥3-fold higher expression during the night, and eight exhibited ≥3-fold higher expression during the day (Table 3). Three of these genes (*AmCry1, AmCry2, Hes/Hey-like)* also exhibited circadian expression patterns in the *A. millepora* microarray. In larvae, both *AmCry1* and *AmCry2* were expressed at higher levels during the day, as they were in the microarray study of adult corals. *Hes/Hey-like* was also expressed most highly during the day in coral larvae. Comparison of the larval dataset with the *Nematostella* DCGs revealed additional shared genes that were not identified in the microarray study. For example, *Clock* expression in larvae was about four times higher during the day compared with the night. A putative homolog of the *clock-interacting circadian pacemaker (CIPC)* exhibited greatly elevated expression during the night in *A. millepora* larvae and also exhibited peak expression during the night in *Nematostella.* CIPC is a mammalian protein that regulates period length by forming complexes with CLOCK, leading to enhanced phosphorylation and degradation^27^,^28^. Although *CIPC* was initially described as absent from invertebrates, similar predicted protein sequences are present in urchins and molluscs (e.g., XP005109657 *Aplysia californica* and XP800566 *Strongylocentrotus purpuratus*). It is unknown whether the CIPC-like protein from *Nematostella* or other invertebrates forms complexes with CLOCK and/or performs a circadian function. Interestingly, in contrast to adult *A. millepora,* the larvae did not show significant (≥3-fold) transcription change between day and night in any chaperone homologues, this may be related to the fact that in the larvae was sampled only once during the daytime and once during the nighttime (ZT10 and ZT22, respectively).

### *Nematostella Cyp17*- and *Cyp21-like* genes have a phase-shifted diel oscillation

Two transcripts belonging to the superfamily of cytochrome P450 mono-oxygenases (CYPs) exhibited diel periodicity in expression, but with a 4–8 hour phase-shift from one another (Fig. 7c). The difference in timing might indicate that the two enzymes catalyze different steps within a metabolic pathway, producing metabolites that cycle out of phase with one another. In a phylogenetic analysis of animal CYPs, these two *Nematostella* CYPs fell into a clade that included the vertebrate steroidogenic *Cyp17* and *Cyp21* genes^29^. Synthesis of vertebrate-type steroids requires side-chain cleavage of cholesterol by the vertebrate-specific CYP11; CYP17 and CYP21 then act catalyze downstream steps in the synthesis of sex steroids and corticosteroids^30^. Several mammalian CYPs, including *Cyp17*, exhibit circadian oscillations in expression, which result in daily cycles in cholesterol homeostasis and hormone concentrations^31^,^32^. While the substrate of the *Nematostella Cyp17*-like genes is unknown, mammalian CYP17 is able to metabolize a variety of substrates including the steroid precursor squalling^33^.

### Profiling of *Nematostella* reveals genes not previously implicated in cnidarian transcriptional oscillations

Several *Nematostella* transcripts exhibited strong diel oscillations that had not previously been implicated in cnidarian circadian signaling. Among these, a transcript (NV_200090) similar to *QN1* (*Centrosomal protein quail neuroretina 1*) exhibited strong cycling with peak expression at night (Supplementary Table S1, Fig 6). In vertebrates QN1 helps to regulate the cell cycle during retinal development and serves a motor protein during mitosis^34^. Of the 50 transcripts oscillating with the strongest diel periodicity (Supplementary Table S1, Fig. 6), four were collagen family members. Collagen transcripts undergo circadian cycles in mammalian cartilage^35^, but rhythms in collagen expression have not been previously identified in cnidarians. Also of note, many of these strongly oscillating genes (7 of 50), exhibited no significant similarity to annotated genes, or could only be weakly annotated as possessing a conserved domain (e.g, LEM superfamily member, GIY-YIG superfamily member, ARID domain-containing protein). Clearly a great deal remains to be learned regarding the function of these cyclic genes.

K-means clustering demonstrated that distinct groups of DCGs exhibit peak expression throughout the day and night. As previously mentioned, three chaperone proteins exhibited peak expression during subjective night (Fig. 6, top cluster; Supplementary Table S1, cluster 1). Beyond this grouping, genes with similar apparent functions did not necessarily cluster together. For example the genes identified as likely circadian regulators (Clock, CIPC, Cryptochromes, Hes/Hey-like) are distributed broadly among clusters. Because circadian regulation is characterized by feedback from intersecting transcriptional/translational loops, it makes sense that expression patterns of regulatory components will be offset. The four collagen-like DCGs were distributed among three expression clusters. While the reason for this offset is unknown, it’s possible that serial expression of different collagen forms helps to stabilize total collagen levels or that the different forms are necessary for specific components that are produced during on a daily cycle.

## Discussion

Through the use of locomotor activity tracking, pharmacological manipulations and transcriptional profiling, we have demonstrated that *Nematostella* maintains a circadian behavioral cycle, revealed a likely role for CK1 in circadian regulation, and identified novel genes with a diel transcriptional cycle.

The automated locomotor activity tracking approach used in this study provides high spatial and temporal resolution. We found that the use of gray scale analysis with an average center point recorded every second was very informative in this study because *Nematostella* exhibited frequent peristaltic contractions and bending movements that often resulted in little or no net distance advancement. These movement types may be missed during still image analysis since single frames are unlikely to capture small repetitive changes.

Our locomotor activity recordings indicate, in accordance with a previous report^16^, that *Nematostella* is a nocturnal animal with daily oscillations in activity that are controlled by an endogenous clock. However, in contrast to previous observation^16^, we found that light completely inhibits *Nematostella* locomotor rhythmicity as no rhythmicity was identified under LL free-run conditions and rhythmicity was lost in response to a light pulse under DD free-run conditions. This difference in results may be due to differences in *Nematostella* populations used, the light and incubation conditions, or the method of recording. Our work also demonstrates that the clock exhibits phase advance in response to a dark pulse during the entrained light period. It also points to the ability of *Nematostella* to maintain a consistent behavioral oscillation period under LD conditions at two different temperatures (18 and 23 °C). This observation suggests that *Nematostella* behavioral rhythms exhibit temperature compensation within a 5 °C range. Temperature compensation is an important feature that corrects for the natural tendency of biochemical reaction rates to change with temperature and thus permits the clock mechanism to have the necessary flexibility to accurately maintain time under changing environmental conditions. The ability to maintain the clock periodicity by compensating temperature is especially important due the rapid climate change and global warming influencing aquatic and marine organisms.

The light level used in our behavioral experiments (200 lux) is low relative to light levels *Nematostella* could naturally experience. At the sediment water interface in Sippewissett Marsh, MA, a site with a natural *Nematostella* population, we frequently measure levels above 20,000 lux (Tarrant, unpublished data). It is difficult to know exactly how *Nematostella* perceives the light environment because the animals are able to burrow into the sediments, which would greatly attenuate their exposure to light. Temperature also produces strong daily cycles in tidepool environments, fluctuating by as much as 20 °C within a single day in Sippewissett Marsh. In addition, *Nematostella* experiences tidal cycles that affect temperature, salinity, oxygen content and prey availability. It is currently unknown which of these potential zeitgebers act to entrain the endogenous clock within natural environments or how these multiple entraining factors may interact.

In *Nematostella* we identified 6 members of the casein kinase I (CK1) family of serine/threonine kinases. Several members of this family have been shown to regulate circadian timing in model organisms through phosphorylation of target proteins, including PERIOD (PER) in bilaterians and FREQUENCY (FRQ) in *Neurospora*^36^. In bilaterian animals, the CK1 clade containing *CK1δ* and *CK1ε* (vertebrates) and *Doubletime* (*Drosophila*) plays a well-documented role in clock function^24^,^37^,^38^. CK1ε regulates the circadian negative feedback loop by periodically binding to and phosphorylating the PERIOD proteins, which form complexes with cryptochromes and regulate transcription by the CLOCK/BMAL1 heterodimer. CK1ε can also phosphorylate other circadian proteins including BMAL1 and cryptochromes^39^. In the golden hamster, mutation of *CK1δ* (*tau* mutant) is associated with a shortened behavioral cycle^40^. In *Drosophila*, mutations in *doubletime* (DBT) alter both behavioral rhythmicity and molecular oscillation through interaction with PER proteins^38^.

We have shown that incubation of *Nematostella* with a pharmacological inhibitor of CK1δ/ε (PF-670462) signaling disrupts the free-running behavioral rhythm. The same treatment with a CK1δ specific inhibitor resulted in no behavioral rhythm change. In bilaterian animals, CK1-mediated phosphorylation of clock components, especially of PERIOD proteins, helps to regulate circadian period. In studies conducted in mammalian systems, PF-670462 exposure resulted in phase shifts or changes in circadian period^41^,^42^. However, similar to our observations with *Nematostella* complete loss of circadian cycling has been observed in zebrafish following exposure to PF-670462^43^; the reasons for these differences among studies and model organisms are unknown. *Nematostella* contains multiple CK1 isoforms, none of which are orthologous to mammalian CK1δ or CK1ε, so it is not clear which form or forms the inhibitor directly targets. Thus, we can only hypothesize that a CK1 family member targeted by the CK1δ/ε inhibitor may be involved in circadian regulation in *Nematostella*, although a potential toxic effect of the CK1δ/ε inhibitor cannot be ruled out. The CK1δ inhibitor is more specific in its targeting of mammalian CK1 genes, and it appears none of the genes regulating circadian behavior in *Nematostella* are sufficiently similar to mammalian CK1δ to be affected by the inhibitor. Also, since homologues of *period* genes have not been identified in *Nematostella* or other cnidarians, it is difficult to predict the targets for CK1 activity although our behavioral data showed arrhythmicity in the presence of the inhibitor and full recovery in the absence of the inhibitor, as found in studies with other model organisms (e.g.^22^,^42^,^43^).

Through high-throughput sequencing, we identified a subset of genes that exhibited diel variation in transcript expression. These included transcripts such as *Clock a*nd cryptochromes that have been identified in previous studies^15^,^44^. Others, like *CIPC* and bHLH-O genes, have well-described roles in bilaterian circadian regulation. Genes in these groups (*CIPC-like* and *Hes/Hey-like*) exhibited daily oscillations both in the present study and in one or more studies of *A. millepora;* however, these genes have not been explicitly discussed as potential regulatory components of the cnidarian clock. bHLH-O proteins generally serve as transcriptional repressors in bilaterians to regulate diverse processes including neurogenesis, vasculogenesis and segmentation^45^. In *Drosophila,* the bHLH-O protein CWO (clockwork orange, mammalian homologues DEC1 and DEC2) competitively binds E-box regulatory elements to modulate CLOCK activity^46^. Similarly, in mammalian systems, HES1 modules CLOCK activity by binding E-box like clock-related elements (EL-boxes)^47^. Thus, we hypothesize that in *Nematostella* HES/HEY-like competitively binds to E-boxes and other regulatory elements to modulate signaling by CLOCK and CYCLE. Because CIPC regulates phosphorylation and degradation of mammalian CLOCK^27^,^28^, we further hypothesize that the *Nematostella* CIPC-like protein also forms complexes with CLOCK and affects its phosphorylation status.

A heme binding gene in the SOUL family and a HMGB gene also exhibited diel cycles both in *Nematostella* and *Acropora;* members of both of these gene families exhibit circadian cycles in other organism, but they are not known to act as core circadian regulators. Heme-binding genes in the SOUL family were originally identified in a screen for genes that were specifically expressed in the chicken retina and pineal gland, two tissues strongly entrained to circadian rhythms^48^. In vertebrates, heme plays an important role in circadian regulation through signaling by the *Rev-erb* nuclear receptors^49^. However, *Rev-erb* homologs are not found in cnidarians, and the role of heme, if any, in cnidarian circadian regulation is unknown. High mobility group B (HMGB) proteins act as DNA chaperones to facilitate complex formation between DNA and proteins including repair enzymes and transcription factors^50^. Circadian expression of some HMGB proteins has been observed in both plants^51^ and animals^52^, and they have been proposed to play a role in temperature compensation^53^.

Transcripts corresponding to chaperone proteins in the Hsp90, Hsp70 and disulfide isomerase families also show consistent daily oscillations in expression in both adult corals and *Nematostella*. Peak expression of these transcripts in late afternoon in the coral *A. millepora* has previously been attributed to defense against oxidative stress related to photosynthesis by symbionts in the corals^20^. Because daily transcriptional patterns in corals reflect the emergent physiology of the host and symbiont (i.e., the ‘holobiont’), interpreting patterns in *Nematostella* can be less complicated. Our observations in *Nematostella* suggest that cycles in chaperone expression may be more fundamentally rooted in circadian regulation. Indeed, studies in mammalian models suggest that some Hsp90 isoforms regulate BMAL1 cellular protein levels^54^, and heat shock proteins have been implicated in both the entrainment and output of the central oscillator^55^,^56^.

In conclusion, this work integrates behavioral studies with transcriptional profiling to investigate the circadian clock of *Nematostella*, a cnidarians species which arose about 700 million years ago^11^. Features shared between the circadian clocks of *Nematostella* and bilaterian animals were most likely present in the earliest metazoans. Our findings show that *Nematostella* meets all major conditions for the function of a true endogenous clock, and can serve as a valuable model organism to study the evolution of animal circadian clock and to understand its function in the cnidarian lineage.

## Materials and methods

### *Nematostella* culture

Laboratory-bred *Nematostella* were maintained in plastic containers with one-third strength artificial sea water (33% ASW, Reef crystals) at 18 °C under a 12 : 12 h (7 am–7 pm/7 pm–7 am) LD cycle. Animals were fed five times per week with freshly-hatched brine shrimp, and water was renewed weekly. Animals were gradually acclimated to 23 °C and starved for two days prior to behavioral experiments and transcriptional profiling.

### Behavioral assays

Locomotor activity of individual Nematostella were monitored using two Noldus DanioVision XT tracking devices, each equipped with an IR camera and white LED illumination that can be set to different intensities and LD cycles (Fig. 1). The data collection and analysis were carried out by EthoVision XT8 video tracking software (Noldus information technology, Wageningen, Netherlands). Animals were isolated in wells of six-well plates, each of which was manually defined as a tracking ‘arena’ in the EthoVision software. Center-point detection with gray scaling (detection range of 25–77, contour erosion of 1 pixel, high pixel smoothing) was used to monitor movements, which were calculated according to the change in position of the average center pixel each second (Fig. 1).

Illumination was provided within the DanioVision tracking device by the integral white LED light with an intensity of 200 (+/−10) lux (25% of its maximum intensity) and did not significantly affect the experimental temperature (23 °C). When needed (as for the 18 °C experiment), a chiller pump was used to keep the water temperature fixed during the duration of the experiment. The illumination cycles were the same as used for culturing (12 : 12 h LD). Since this is the first application of this tracking system to measurement of sea anemone movements, we tested the system background noise using measurements of six immobilized (paralyzed with MgCl_2_) *Nematostella* individuals for 1 h. The recorded movement in this test was less than 1 cm, and was considered as insignificant background noise (compared with the average movement of the non-paralyzed animals). Parameters were optimized to ensure that organisms were detected throughout the entire observation period.

### Locomotor activity data analysis

The total distance moved was summed in hourly bins and expressed as a percentage of the maximum hourly distance measured for each individual. The average and standard errors were calculated for all tested animals based on the normalized values of each hour. The oscillation frequencies were evaluated based on the average values of each experiment using Fourier analysis, as previously described^57^.

### Casein Kinase inhibition

*Nematostella* individuals were monitored in 6-well plates containing one-third strength ASW with one of two casein kinase inhibitors; the pan-CK1δ/ε inhibitor PF-670462 or the CK1δ-selective inhibitor PF-4800567 (Pfizer Global Research and Development, TOCRIS Bioscience) dissolved in DMSO. In order to determine the effective concentrations, we performed an initial toxicity assay based on the range tested by Smadja Storz *et al.*^24^. We tested the viability of the animals based on response to mechanical touch 1, 3 and 10 days after adding the inhibitors to the water in the six-well plates to final concentrations of 0.1, 1 and 10 μM (n = 6). All *Nematostella* individuals survived up to 10 days after incubation in 0.1 and 1 μM of both inhibitors, but all died 10 days after incubation in 10 μM concentration of either inhibitor. Based on these results, all inhibition experiments were conducted in final concentrations of 1 μM. All controls were treated with identical concentrations of DMSO (0.05%).

### RNA-seq

We used RNA-seq technology to identify diel cycle genes (DCGs) in *Nematostella* following an experimental design previously used in circadian studies of the coral *Acropora millepora*^20^. Anemones were acclimated and maintained during the experiment inside the Noldus DanioVision XT tracking device under identical light (LD) and temperature conditions as in the behavioral assay. Five anemones were sampled every 4 h over two consecutive days, starting at 8 am. Total RNA was extracted from pools of five individuals using the Qiagen RNeasy Mini Kit. The Illumina TruSeq protocol was used to prepare libraries from the RNA samples. We performed one biological replicate by constructing and sequencing two Illumina libraries from different samples of five animals collected at same time point (the second time point, 12pm). The libraries were multiplexed on 2 lanes of an Illumina HiSeq2000. On average, ~15 million 50 base-pair paired-end reads were obtained for each library. The data was deposited as an SRA BioProject (accession number: PRJNA246707). Reads were aligned to the *Nematostella* genome^11^ using TopHat^58^. Only reads that uniquely aligned to protein coding regions with up to two mismatches were retained. The *Nematostella* gene information was downloaded from Joint Genome Institute database ( http://genome.jgi-psf.org/Nemve1/Nemve1.info.html). A custom Perl script was used to parse the output from TopHat (Sequence Alignment/Map (SAM) format) and to convert it into raw number of reads aligned to each position in each *Nematostella* gene. The dataset was de-duplicated to remove multiple reads with identical start positions in the genome, as these might represent PCR artifacts^59^. Library quality was assessed in comparison with a benchmark library described by Levin and colleagues^59^. All library quality parameters met the benchmark standards, including mapping of reads to unique genome start sites and evenness in expressed gene coverage.

We tested the effect of biological variation by comparing two libraries derived from different anemones collected at the same time and light condition. The differences between the samples are close to the expected technical noise (96% of the genes are within the expected 99%-region of Poisson noise), as described recently for miRNA-seq multiplexing^60^.

The logarithmically-transformed gene expression values were normalized using a modification of the TMM method^61^, in which the mRNA profiles were scaled such that the log-fold changes of all the mRNAs are distributed around zero (after trimming the higher and lower quartiles of the log-fold changes). The scaling factor was thus set so that the trimmed mean of log-folds vanish. The mean was weighted using the inverse standard deviation, as estimated from Poisson distribution of counts^60^.

### Fourier analysis for expression pattern

The time-dependent signal was converted into a frequency-dependent signal using the Fast Fourier Transform (FFT). We used in-house scripts that were previously found to be accurate in detecting circadian genes, as attested by ~90% true positive rate in independent validation experiments (20,62). The extent to which the original signal contains a 24-hr rhythm was quantified by the ratio (‘g-factor’) of the power (squared amplitude) of the frequency which corresponds to a 24-h period, to the sum of powers of all frequencies. The higher the g-factor, the higher is the confidence that the transcript exhibits a diel rhythm. Changing the definition of the g-factor by adding the powers of higher harmonics of the 24-h period to the numerator, gave similar results compared to the use of the definition above. The genes with the highest g-factor (g-factor greater than 0.5 was used as a cutoff) were sorted into five clusters with similar temporal expression patterns using a K-means clustering, implemented in Matlab as described by Levy *et al.*^20^.

### Annotation of DCGs

Functional annotation of *Nematostella* transcripts, including predicted homologs within the Swissprot database and from the transcriptome of the coral *Acropora millepora* were downloaded from the Joint Genome Institute database. Annotations were manually curated for genes exhibiting strong diel periodicity in their expression patterns (50 genes with highest g-factor) and those identified through our comparative analysis (see below). Manual curation was based on BLASTp searches of the Swissprot and NR databases and, in a few cases, published phylogenetic analyses (cryptochromes, *Hes*/*Hey-like*).

### Comparative transcriptomics

We compared the set of DCGs identified in our study with genes exhibiting circadian expression patterns or strong day/night differences in two published studies of the coral *Acropora millepora.* Brady *et al.*^19^ used Illumina-based transcriptional profiling to compare gene expression between coral larvae collected during day and night (12 : 12 h LD cycle, samples collected 10 hours after lights on (ZT10) and 10 hours after lights off (ZT22)). They reported the number of counts and fold change, but did not provide any further statistical analysis. From the 47,666 transcripts that they identified, we selected the 10,294 genes that exhibited a three-fold difference in expression between the day and night and identified potential homologs of the putative DCGs from *Nematostella.* Levy *et al.*^20^ used an experimental design similar to the present study: *Acropora millepora* colonies were sampled every 4 hours over 2 days under LD and DD conditions. They conducted expression profiling using a cDNA microarray. We selected 200 genes exhibiting the strongest circadian expression patterns (g-factor > 0.6468), identified the associated probe sequences in the NCBI Gene Expression Omnibus (GEO) database (Platform GPL6941), and annotated them using BLAST searches of a *Acropora millepora* larval transcriptome database hosted on SymBioSys (http: sequoia.ucmerced.edu/SymBioSys). We then identified potential homologs among the putative DCGs from *Nematostella*.

## Additional Information

**How to cite this article**: Oren, M. *et al.* Profiling molecular and behavioral circadian rhythms in the non-symbiotic sea anemone Nematostella vectensis. *Sci. Rep.* **5**, 11418; doi: 10.1038/srep11418 (2015).

## Supplementary Material

Supplementary Information

Supplementary Data Set 1

## Figures and Tables

**Table 1:** Comparison of peak expression times of selected core clock genes between *Nematostella* and *Acropora millepora*. All experiments were conducted using a 12 : 12 h light : dark cycle. Peak expression indicated as Zeitgeber time (ZT), which in this case indicates the number of hours after the lights were turned on. Data from qPCR^15,19^, Illumina (this study), and microarray^20^.

***Nematostella vectensis***				***Acropora millepora***		
Gene	Accession numbers	Reitzel *et al.* 2010	This study	Accession numbers	Levy *et al.* 2011	Brady *et al.* 2011
*Clock*	JGI: 160110 XP_001639742	ZT11	ZT9-13	None/Unknown	NA	ZT14
*NvCry1a*	JGI: 168581 XP_001631029	ZT4-ZT11	ZT5	CRYb (DY585180; SeqIndex10300; probe A031-G12)	ZT5	NA
*NvCry1b*	JGI: 16062 XP_001632849	ZT7	ZT5-9	CRY2 (EF202590; SeqIndex 10301)	NA	ZT2
*NvCry2*	JGI: 194898 XP_001623146	ZT0-4	ZT21	CRY1 (EF202589; SeqIndex 10302, probes C018-C3, D027-B12)	ZT5	ZT6

**Table 2:** Homologous gene pairs exhibiting light-entrained diel expression cycles in both *Nematostella* (present study) and *Acropora millepora*^20^.

**Gene number**	**g-factor**	**Annotation**	**Peak**	**Acropora SeqIndex**	**Acropora peak (# probes)**
246249	0.9231	*Hes/Hey-like*	12 pm	18661	8 am (1/1)
185184	0.8102	*High mobility group protein B3*	12 pm	7362	4 am (1/1)
114661	0.8014	*Heme-binding protein 2* (SOUL family)	4 pm	16238	12 pm (30/32)
181671	0.7892	*Heat shock protein 108* (Hsp90 family)	12 am	2404	4 pm (5/5)
167250	0.7128	No hits	8 am	18512	8 pm (2/3)
193399	0.692	*Protein disulfide-isomerase A4*	12 am	2399	4 pm (1/1)
216823	0.6703	*78 kDa glucose-regulated protein* (Hsp70 family)	12 am	12749	4 pm (2/2)
194898*	0.5128	*Cryptochrome 2*	4 am	10302 (Cry1)	12 pm (2/2)
168581*	0.5076	*Cryptochrome 1a*	12 pm	10301 (Cryb)	12 pm (1/1)

Annotations are based on BLASTp results of the Swissprot database as well as phylogenetic analyses of cryptochromes^17^ and basic helix-loop-helix genes i.e., Hes/Hey-like;^26^. Columns labeled “peak” indicate times of maximum expression. In many cases, multiple microarray probes corresponded to a single SeqIndex. The fraction of probes showing the described circadian pattern is indicated parenthetically.

**Table 3:** Homologous genes exhibiting daily variation in expression both in the present study of *Nematostella* and in a study of *Acropora millepora* larvae. See text for additional details. Peak refers to the time of maximal expression within the *Nematostella* study.

**Gene number**	**g-factor**	**Annotation**	**Peak**	**Acropora SeqIndex**	**Day/Night Counts**
Homologues of genes upregulated during day in coral larvae					
246249	0.9231	*Hes/Hey-like*	12 pm	18661	407/90
160110	0.7001	*Clock*	8 pm	10199	681/216
241935	0.5782	*Signal transducer and activator of transcription 5A*	8 am	70856	9/3
243788	0.5448	*Pleckstrin homology domain-containing family G member 5*	12 pm	61779	50/14
194898*	0.5128	*Cryptochrome (NvCry2, AmCry1)*	4 am	10302	5724/353
168581*	0.5076	*Cryptochrome (NvCry1a; AmCRYb)*	12 pm	10301	1843/309
Homologues of genes upregulated during night in coral larvae					
245026	0.9255	*Clock-interacting circadian pacemaker (CIPC)*	4 am	90172	7/181
167250	0.7128	*No hits*	8 am	18512	98/1513
163545	0.636	*Transcription factor Sox17αB)*	8 pm	3863	36/449
240625	0.6005	*NIPA-like protein*	12 pm	10904	2/7
242499	0.5467	*Sortilin-related receptor*	4 pm	13077	2/9
212997	0.5288	*Protein BZZ1*	12 pm	18943	1/3
192745	0.5265	*Putative adenosyl-homocysteinase 3*	12 p m	13339	2/14
98402	0.5059	*Conserved oligomeric Golgi complex subunit 5 (COG5)*	4 pm	15726	4/18

Day/Night counts refers to the number of counts mapped to a given gene in *Acropora millepora* larvae sampled during the day and night, respectively.
