# Supplementary material for: Profiling molecular and behavioral circadian rhythms in the non-symbiotic sea anemone Nematostella vectensis
Source: Sci Rep. 2015 Jun 17;5:11418. doi: 10.1038/srep11418 (PMC4476465; doi:10.1038/srep11418)
Supplement: Supplementary Information [file srep11418-s1.pdf]

Profiling molecular and behavioral circadian rhythms in the non-symbiotic sea anemone *Nematostella vectensis*

Matan Oren[1*#], Ann M. Tarrant[2#], Shahar Alon[3,4], Noa Simon-Blecher[1], Idan Elbaz[1,5], Lior Appelbaum[1,5], Oren Levy[1*]

[1] The Mina & Everard Goodman Faculty of Life Sciences, Bar-Ilan University, Ramat-Gan 52900, Israel

[2] Biology Department, Woods Hole Oceanographic Institution, Woods Hole MA 02543, USA

[3] George S. Wise Faculty of Life Sciences, Department of Neurobiology, Tel-Aviv University, Tel-Aviv 69978, Israel,

[4] Sagol School of Neuroscience, Tel-Aviv University, Tel-Aviv 69978, Israel,

[5] The Leslie and Susan Gonda Multidisciplinary Brain Research Center, Bar-Ilan University, Ramat-Gan, Israel

*Corresponding authors: Matan Oren & Oren Levy

e-mail: matanok@gmail.com; oren.levy@biu.ac.il

# These authors contributed equally in this work.

Supplementary Figure S1

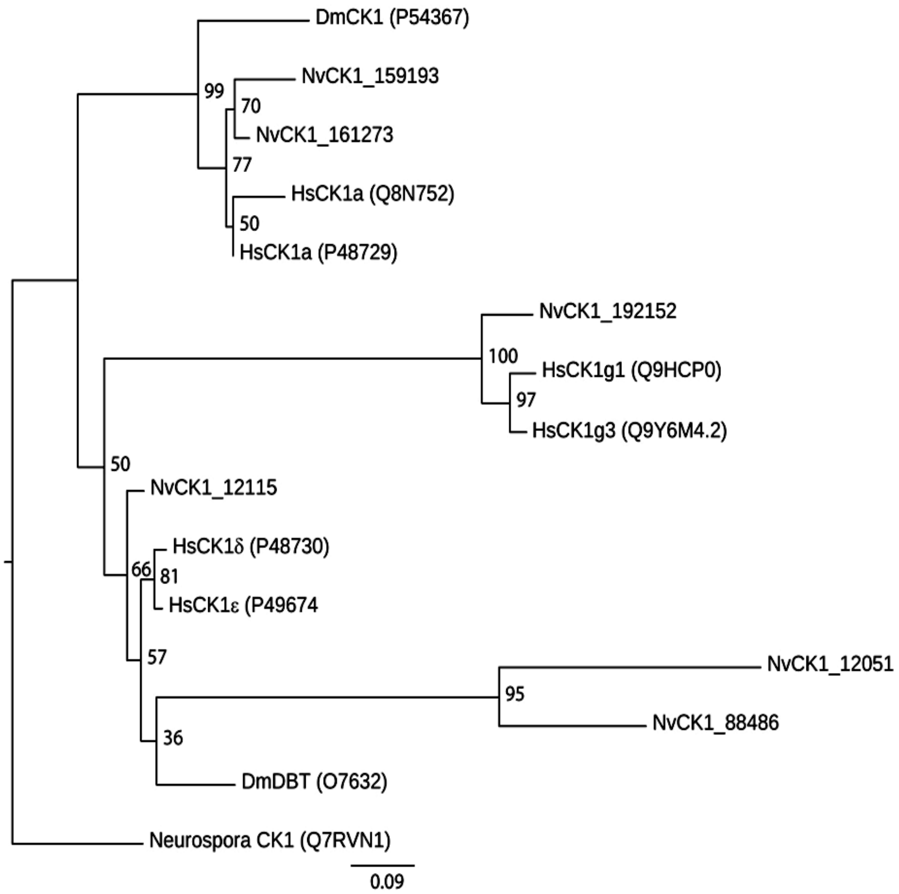

Phylogenetic tree showing relationships among CK1 genes from *Drosophila melanogaster* (Dm), *Homo sapiens* (Hs), *Nematostella vectensis* (Nv), and *Neurospora crassa* (Neurospora). Sequences were aligned using Muscle [1], and a likelihood-based tree constructed with the WAG+G+F substitution model using RAxML [2]. Numbers on nodes represent the percentage of 1000 replicates supporting a given grouping Genbank accession numbers given parenthetically. For *Nematostella* sequences, JGI accession numbers are given following the underscore.

References

1. Edgar, R.C. MUSCLE: multiple sequence alignment with high accuracy and high throughput. *Nucleic Acids Res* 2004, **32**(5):1792-1797.
2. Stamatakis, A. RAxML-VI-HPC: maximum likelihood-based phylogenetic analyses with thousands of taxa and mixed models. *Bioinformatics* 2006, **22**:2688-2690.
